# Observations on the Use of Opium in Removing Symptoms Supposed to Be Owing to Morbid Irritability

**Published:** 1785

**Authors:** 


### THE LONDON MEDICAL JOURNAL,

### FOR THE YEAR 1785,

### PART THE FIRST.

### SECTION I.

### ESSAYS AND OBSERVATIONS.

I. *Obſervations on the uſe of Opium in removing ſymptoms ſuppoſed to be owing to Morbid Irritability. Communicated in a letter to Dr.* Simmons, *by Mr.* Alexander Grant, *ſenior Surgeon of His Majeſty's Military Hoſpitals during the late war in North America.*

IN the year 1779 I had an opportunity of obſerving, in His Majeſty's General Hoſpital at New York, the good effects of Opium, in a variety of caſes originally Venereal, in which mercurial preparations appeared to be of no efficacy.

It happened that a great number of ſoldiers came under my care at the ſame time, who laboured under evident ſymptoms of morbid Irritability, which might be owing to different

 cauſes caufes; but all thefe patients *were undergoing*, or *had undergone* a courfe of mercury.

Thefe fymptoms, in all their variety of appearances and changes, at firft arifing from the venereal virus, and terminating in what has fince, upon reafoning and reflection, appeared to me to have been brought on by morbid Irritability, were all relieved, and finally the cure compleated, by alleviating pain, and procuring reft.

And here I may remark, that foldiers in general are cured of the Venereal Difeafe with more difficulty than any other Patients, owing to their ignorance and inattention, or to their defire of protracting the difeafe, and fruftrating the ufual modes of relief.

The ftate of the difeafe in which (as far as I am able to judge from the trials I have made) our expectations are likely to be anfwered from the ufe of Opium, is when the ulcers are fpreading, or continue much the fame, with a foul appearance, bad difcharge, and much pain, the patient having little or no reft, and all local applications feeming to be of no fervice: to thefe fymptoms may be added a quick pulfe, which is an invariable attendant, being, in general above an hundred, fometimes an hundred dred and twenty, and ſometimes more, in a minute.

When I firſt adminiſtered Opium in caſes of this ſort, I had the ſatisfaction to find, that I gained two material points, viz. Eaſe and Reſt; with ſome in the firſt twenty-four hours, and in general within two days; afterwards, from theſe good conſequences, I was induced to continue the uſe of it, increaſing or diminiſhing the quantity as occaſion required, and entirely omitting at the ſame time the uſe of mercurial applications, either externally or internally.

Without entering into a diſquiſition on Opium, I take the liberty of concluding, that it will be ſufficient if I am enabled to ſtate facts, and mention the uſeful effects of this medicine in a diſeaſe, which is one of the moſt violent that the human body can be afflicted with.

With regard to the action of Opium on the body in this diſeaſe, I have to obſerve, that in ſome patients its effects were ſudden, in others gradual; in general the circulation of the blood was very much decreaſed by it, and ſometimes the pulſe was rendered ſo ſlow as to beat only fifty and forty times in a minute; but this effect is not always neceſſary to the cure, as a good deal will depend upon the patient's diſpoſition to Irritability, which is the principal thing I attend to—for I have conſtantly obſerved, that as ſoon as that has been leſſened, ſome ſalutary change has been produced on the ſurface of the ulcer.

Its effects, in a few of the caſes, were almoſt inſtantaneous; amongſt theſe was that of a young man, twenty-two years of age, who had two Buboes (one in each Groin) which were opened and treated in the uſual way, but continued in an ulcerated ſtate for five months, during which time he rubbed in three ounces and a half of ſtrong mercurial ointment, which ſeemed ſufficiently to affect the conſtitution; a proper diet was uſed, and every thing elſe directed which was thought neceſſary at the time. No change being perceived, he was ordered to waſh out, and take purging phyſic, which he did ſeveral times. Three weeks elapſed, and at this period he was not in the leaſt emaciated, but remained in full ſtrength and good habit of body. At this time the ulcers were foul, with thick edges, and the diſcharge from them ichorous, with conſiderable pain; and the patient had little or no reſt. I then began with Opium, and gave him the firſt night one grain grain and a half only, directed a low diet, and dreſſed the ulcers in the moſt ſimple manner; the next day there was no alteration: I increaſed the doſe of the medicine, half a grain that day; the following day he was much the ſame; but the day afterwards, which was the fourth, a favourable change was obſerved; and, from that time to the expiration of three weeks, gradual changes took place, and both ulcers perfectly healed, without his having had occaſion to increaſe the doſe, or to make uſe of any other medicine. And here I will beg leave to remark, that with every patient I have avoided, as much as poſſible, ſuch applications as ſeemed to make the parts in any way uneaſy. A well-made bread and milk poultice will anſwer in the early part of the treatment, and afterwards dry lint and cerate; but, if a fungus exiſts, I prefer a ſolution of thebaic extract in an oatmeal poultice, applied cold, in contact with the ſore, to any thing elſe.

The manner in which the alteration takes place is both curious and ſingular; the firſt favourable ſymptoms are eaſe and reſt; the next, if I may be allowed the expreſſion, is an unfolding or looſening of the texture of the ſurface of the ulcer after the irritability is leſſened, which effect ſoon takes place: the parts are in a ſtate of relaxation, owing to the ſtricture on the ſurface being relieved, and finally the cure is aſſiſted by the ſkin corrugating, or contracting to the center of the ulcer, unaided by ſtimulating applications, and, frequently, with very little aſſiſtance by the uſual way of a new ſkin.

In ſeveral caſes I have obſerved the cure go on extremely well, without the ſurface of the ulcer appearing florid at any one time, as is uſual in healthy-looking ſores. I am very happy in this part, in having Mr. Wier's, Surgeon of His Majeſty's Military Hoſpitals, permiſſion to ſay, that he has obſerved all the above facts that I mention. And ſince that, having communicated my ideas to Mr. Forſter, who is likewiſe Surgeon of His Majeſty's Military Hoſpitals, and whoſe abilities I reſpect, I have much ſatisfaction in being able to add his opinion in corroborating the above facts; Mr. Forſter having obſerved the ſame appearances and changes that I have endeavoured to deſcribe.

The third, fourth, or fifth day was the time I in general found the appearance of the ſores change for the better.

The

The dofe that I have in general begun with in this difeafe, has been about one grain and a half the firft night, which I have increafed night and morning, until I found it anfwered the intended purpofes. With a view of keeping the patient quiet, and lefs difpofed to move, I have always divided the time, and therefore have given it night and morning; and what is very extraordinary, the patient in the day-time never appeared to me more drowfy than if he were not making ufe of the medicine; but with fome an indolence took place, and continued, or difcontinued, either as the conftitution became ufed to the medicine, or to the variation of the dofe. I confefs I was always happy when it appeared; as I never knew any harm accrue from it: and, befides its own action internally, Opium difpofes a patient of a reftlefs temper of mind, to obferve a proper regimen, and to obey any directions that may be thought neceffary at the time. I have always obferved, that it is beft to keep the patient ignorant of the medicine whilft he is ufing it.

A tremor, which fometimes came on, never obliged me to decreafe the dofe, or to pay any particular attention to this fymptom, unlefs the bowels were difpofed to be coftive; and

 then then a purge readily relieved it in a great meaſure.

The preparation that I invariably gave my patients was *Extractum Thebaicum*; if any objection aroſe to its being uſed in a ſolid ſtate, I preferred diſſolving the doſe in plain water to the tincture, or any other form.

With regard to the quantity that may be given, I will not determine; I think for the moſt part the diſeaſe yielded to about four or ſix grains a day; I have in two or three caſes given eight grains, and in one extraordinary caſe of a cancerous lip, of near three years ſtanding, I increaſed the quantity to twenty-four grains in a day, divided into three doſes. The patient was a man about fifty years of age, and I was ſix weeks in bringing him to this doſe, which I continued for a few days, and finding that it did not do any material ſervice in the progreſs of the cure, I diſcontinued it; yet now and then a favourable change ſeemed to appear, for two ſinuſes, that I had occaſion to open, digeſted well, and cloſed; no ill effects were produced by the medicine, excepting ſometimes a giddineſs, which was always relieved by a gentle purge. Previous to his making uſe of Opium, he had always a quick pulſe; but it afterwards decreaſed to between forty and fifty ſtrokes in a minute, and continued ſo for ſome time. And I cannot help being very much of opinion, that had Opium been properly given in the earlier ſtate of the diſeaſe, a cure might have been obtained. In this opinion I am confirmed by a caſe I have lately attended, which was an ulcer in the mouth, of three months ſtanding, with all the characteriſtic marks and ſymptoms of an incipient Cancer, and which was completely cured by Opium and Cicuta joined. This patient (Major Sinclair) Dr. Garthſhore attended with me; the firſt time we ſaw him we agreed in opinion with reſpect to the means to be purſued for his cure, and never had occaſion to deviate from the plan. Before he began the uſe of theſe medicines, he was almoſt in a ſtate of diſtraction from pain, and his pulſe beat an hundred and twenty ſtrokes in a minute. This quickneſs of pulſe was diminiſhed on the third day, and about the tenth the ulcers changed for the better; and in a month afterwards he was quite well *.

* This Patient drank a decoction of four ounces of Sarſaparilla every twenty-four hours during his cure.

Gentle Purges frequently become neceffary in the early part of ufing the medicine, though given only in a fmall dofe, efpecially where the body is difpofed to be coftive; and I have generally found that a little Glauber's Salt anfwered the purpofe, as this operated without my being obliged to delay the ufe of Opium. On the fecond or third day with fome a head-ach came on in confequence of the bowels being conftipated; but this always readily gave way to a gentle purge; and I have always found, that when the conftitution became ufed to the medicine, the pain of the head was no longer an attendant, and in general the body became regular; nay, with fome a Diarrhœa has been produced, which has continued for two or three days, and that without feeming to do any harm.

An increafed fecretion of faliva is not uncommon, as well as of urine; it does not depend upon taking a large quantity, for it will happen fometimes in the firft fix days; but neither the one nor the other feems at any time to be injurious, nor does it appear neceffary to forward the cure, as patients who have experienced neither of thofe effects have obtained it in as fhort a fpace of time.

With

With regard to the diet of the patients, I think it right to obſerve, that until I procured eaſe and reſt, I continued them on a low diet, which I in general began with; and as circumſtances changed afterwards, ſo I made ſuch alterations as the conſtitution ſeemed to require; ſometimes adding meat, and ſometimes either a gill of rum in the day, or wine in proportion.

From the above obſervations it will appear, that I am inclined to confine the good effects of Opium (which are indeed very conſiderable) to an advanced ſtage of the venereal diſeaſe, or what, in my humble opinion, may more properly be termed *Morbid Irritability* ſimply, in which ſtate, mercury has either loſt its efficacy, or ſeems to do miſchief; and this irritability may, I apprehend, probably ariſe from different cauſes, as it certainly may be a queſtion, whether there was any Virus of a venereal nature remaining in the caſes in which I gave Opium: however that may be, the good effects of the medicine were unqueſtionable; and it will appear that I do not truſt to Opium as a ſpecific in recent caſes, or where any part of the Venereal Virus might ſeem to remain; for the laſt caſe I had under my care being of ſo complicated a nature, I joined Mercury with the

Opium

Opium for the laſt three weeks, but not till I had fully and effectually relieved the ſymptoms of Morbid Irritability.

The caſe I allude to was that of a bad ſore throat, which came under my care about ſix months ago. The patient was a gentleman of about three or four and thirty years of age, and of a thin habit.—At the time I firſt ſaw him he was rather in an emaciated ſtate, and was purſuing a courſe of mercury, which he had begun about two months before. I found, upon examination, ſeveral ulcers in the Fauces, and a large one on the left tonſil; he had a ſevere pain on the fore part of one leg, with the Perioſteum thickened — an eruption on his breaſt — his pulſe beating an hundred and thirty ſtrokes in a minute — a moſt reſtleſs diſpoſition — great anxiety — and he had not had any ſleep for ſome weeks. I omitted entirely the whole of the plan he was then upon, and did not give him any thing till bed-time, when he took one grain and a half of Opium, and was directed to repeat one grain the morning following. Upon ſeeing him the next day, before I aſked him a queſtion, he cried out, " I am a well man to what I was; I have had no pain ſince laſt night, and ſlept well." This formed the foundation of his cure. I increaſed the doſe, till he took three grains at night, and two in the morning.

On the ſecond day he made uſe of the ſteam of hot water, through the inhaler, which he repeated occaſionally afterwards with evident advantage.

On the third day the ulcers ſeemed to appear cleaner, and every day afterwards he gradually grew better, till the twenty-fourth day, when all the parts were healed.—On the fifth day I obſerved that his pulſe was in a natural ſtate, and it continued ſo the whole time of his recovery.

On the eighth day of his uſing the Opium a ſpitting came on, and continued eleven days. For a few days he ſpit about a pint each day. This did not weaken him in the leaſt—from being confined to his bed, he was able to walk about, and every day gained ſtrength. Juſt at this time I directed a quart of very ſtrong decoction of Sarſaparilla to be taken every day, which produced a gentle diaphoreſis; and I continued this plan for ſix weeks, and, for the ſake of ſecurity, for the laſt three weeks I joined to the Opium at bed-time half a gain of Mercurius Calcinatus. In the courſe of the firſt fortnight the eruption diſappeared, and the thickened Perioſteum gave way to a bliſter.

On the twentieth day, I began to decreaſe the quantity of Opium; and from the twenty-fourth to the forty-ſecond day, when I left it off, conſidering him as perfectly cured, I only gave him one grain in the twenty-four hours.—He had occaſion to make uſe of purging medicines at different times, owing to a coſtive habit of body; but theſe never interfered with the Opium.

As conciſely as poſſible I will now beg leave to mention the following caſes:

I. William Rockett, of the detachment of Guards that was in America during the late war, thirty-ſeven years of age, had ulcers on each tonſil and almoſt the whole of the fauces, with violent pains in his bones. Theſe complaints were of three months ſtanding; and mercurials and other medicines, had been adminiſtered during the whole of that time.

He was of a thin habit of body, with a pulſe from an hundred and twenty to an hundred and thirty ſtrokes in a minute. He began with one grain and a half of Opium the firſt night.

On the third day, no alteration having taken place, I increafed the dofe one grain.

The next day, to appearance, the ulcers feemed not quite fo angry ; and in every other refpect he was better.

On the eighth day, he appeared weak, and his throat in general relaxed. I omitted the Opium, and began with the Peruvian Bark, in as large dofes as the patient's ftomach would bear, and ordered him to make ufe of an aftringent gargle.

On the tenth day, the bark occafioning naufea, I was obliged to leave it off. The pain in his bones returning with violence, I again gave him two grains of Opium in the morning.

The next day he was better.

On the twelfth day, as he was much the fame, I increafed the dofe of Opium one grain in the morning, and continued this plan to the fifty-fecond day, without varying the dofe ; when every thing feeming to go on well, and the pain of the bones having left him, I omitted the two grains in the morning.

In a week afterwards all the ulcers were healed ; and he was gaining ftrength daily. I continued to give him one grain of Opium at night for a fortnight longer, and difcharged him from the hofpital perfectly cured. It was near a fortnight before the pulfe returned to its natural ftate ; and afterwards it was frequently under fixty.—This cafe happened among the firft in the year 1779: and Mr. Rufh, Surgeon to the Second Troop of Horfe Grenadier Guards, and late Surgeon to the Brigade of Guards in North America, was witnefs to the whole of the treatment.

II. Thomas Wells, thirty-fix years of age, of a ftrong plethoric habit of body, with rather a full pulfe, had fmall ulcers on the tonfils and fauces, and an ophthalmia, for which he had been falivated without the leaft benefit. I directed for him a low diet, and began with giving him one grain of Opium night and morning.

The next day he was eafier.

The fecond day much the fame.

The third day a good deal better.

And on the fixth day, being much the fame, I increafed the dofe of Opium one grain at night.

The next day he was going on well, and continued getting better till the fourteenth day, when his throat was quite well, and his eyes much relieved.

On

On the ſixteenth I omitted the Opium in the morning; and on the eighteenth, as he was nearly well, I omitted one grain at night.

On the twenty-third, the inflammation was gone; and in every other reſpect he was perfectly well.

On the thirtieth day the Opium was entirely omitted; and he was diſcharged from the hoſpital.

III. April 7th, 1782, I was deſired to ſee John Blewers, a ſailor, twenty-eight years of age, of a plethoric habit of body, with ulcers on the glans penis, a phymoſis, the præputium in a gangrenous ſtate, and ſuffering moſt excruciating pain; his pulſe beat an hundred and twenty ſtrokes in a minute, and appeared to be increaſing in quickneſs. He had rubbed in enough of the mercurial ointment to affect his mouth twice.

I began with two grains of Opium at night and one in the morning.

The next day, he ſaid he had enjoyed a good night's reſt, which he had been a ſtranger to for ſome time.

On the ninth he was much the ſame in appearance; and I directed two grains of Opium to be taken night and morning.

On the tenth he had eaſe and reſt.

On the eleventh the pain was much relieved; and there was an appearance of part of the præputium floughing.

On the twelfth he was not quite fo well; and the dofe of Opium was now increafed to three grains night and morning.

On the thirteenth, he was better.

On the fourteenth, the flough was thrown off; and he was better in every refpect.

On the fifteenth, he was in a good ftate, and continued going on well till the eighteenth of May, when the ulcers being nearly healed, I decreafed the dofe of Opium to four grains a day.

On the twentieth and twenty-firft, I decreafed the dofe two grains more.

On the twenty-fifth, he was quite well, but continued to take a fingle grain a day for a week longer.

Befides the above, I had the fatisfaction of effectually relieving, with the fame manner of treatment, ten other patients; of whom, fix had either obftinate or fpreading ulcers in the groin; one an ulcerated glans penis; and three ulcerations of the tonfils and fauces.

Having remarked, that in cafes where fungus exifts, I prefer a folution of thebaic extract tract in an oatmeal-poultice, made and applied cold, I beg leave, as proofs of its peculiar efficacy, to add the following cafes; neither of which, as far as my inquiries went, originated from the Venereal Difeafe.

I. Ifaac Pratt, twenty years of age, had a very large ulcer on the fore part of his right leg, with a fungus at leaft an inch high on one part, inclining to the infide of the leg, and of a hard texture.—This ulcer, which came on after a long intermittent fever, began with an inflammation, attended with fmall white blifters. It grew worfe and worfe, and when I firft faw him was of thirteen months ftanding. The Peruvian Bark, and other medicines, were occafionally made ufe of, as feemed neceffary, as was alfo a variety of external applications (including a tight bandage) without producing any favourable change, though the patient recovered his health in every other refpect. The difcharge from the ulcer was ichorous, and at times the pain was very great; but at intervals he was perfectly eafy, and his pulfe was never affected, excepting when he was in pain.—Conjecturing that Morbid Irritability might be the caufe of the ulcers continuing in the fame bad ftate, I left off the ufual dreffings, and in their ftead

ſtead applied to the ſore an oatmeal-poultice, prepared with a ſolution of thebaic extract, in the proportion of three drachms of the latter to eight ounces of cold water. This dreſſing I renewed twice a day; and I likewiſe began with giving him internally one grain of Opium night and morning.—This was on the 25th of February, 1780.

On the 28th no alteration being produced, I increaſed the doſe to two grains night and morning.

March 3d, no alteration being obſerved, I increaſed the doſe of Opium to three grains night and morning: at this time I found it neceſſary to give him a purge.

On the 5th, there ſeemed to be an appearance of a favourable change.

During the five following days the changes for the better, though ſlow, were very evident; and, upon applying my finger to the fungus, I felt that it was not quite ſo hard as before. At this time another purge was thought neceſſary, and the patient continued till the latter end of April, gradually getting better.

The ſame doſe of Opium, viz. three grains, was conſtantly exhibited; and now and then, as occaſion required, a ſmall doſe of ſalts, which which always anfwered the purpofe of procuring ftools when well diluted.

On the 3d of May the fungus was totally gone, and the ulcer nearly healed.

On the 5th, I decreafed the dofe of Opium to two grains in the day.

On the 7th, I decreafed it to one grain;

And on the 8th I entirely left it off.

On the 20th, the ulcer was perfectly cicatrized. I had continued the poultice till within fix days of the cure, and then applied cerate.

About eight months afterwards, I faw this man, and he informed me, that his leg had continued well from the time he was difcharged from the hofpital.

This ulcer healed without having had at any one time a florid appearance.

This cafe, with others that I have had an opportunity of obferving, together with the remarks of my colleagues, Meffrs. Forfter and Wier, will, I flatter myfelf, be fufficient to make Surgeons lefs fearful of the probability of a cure being obtained, and lefs defirous of ufing ftimulating applications, with a view of producing the change to a florid, or what is commonly termed healthy ftate of an ulcer;

 for, for, as far as I am able to judge, by fo doing the cure will be protracted.

II. March 16th, 1780, Abraham Elftone, forty-one years of age, was received into the General Hofpital, with an ulceration on the whole furface of the chin and left fide of the lower jaw, with a fungus of nearly the thicknefs of half an inch, irritable to the touch, and extending univerfally over the ulcer, and attended with an ichorous difcharge. He had been fix weeks in this ftate—He informed me, that at two different times, in the early part of his life, he had been fubject to an enlargement of the maxillary glands, which had always been foon relieved.—The Surgeon, whofe care he was under for the prefent difeafe, confidered the cafe as fcrophulous, and treated it accordingly, without any fort of benefit, as the ulcer continued to fpread, and was rendered exceedingly painful by the growth of the patient's beard.—I directed one grain of Opium to be adminiftered night and morning, and likewife recommended the ufe of an opiate poultice, cold, as in the former cafe: this method was perfevered in till the twentieth, but without any alteration, although the dofe of Opium was increafed to four grains night and morning.

On

On the 25th, there seemed to be an appearance on the ulcer for the better.—I increased the dose of Opium to five grains night and morning.

From the 25th of March to the 3d of April, the patient complained less of pain: and from the 4th to the 18th of April, the fungus was gradually decreasing.

On the 23d, the discharge was changed for the better, and the fungus was nearly gone;

And on the 25th, there was not the least appearance of fungus remaining, and the parts were perfectly easy and relaxed.

About a fortnight afterwards the ulceration was quite healed, and I laid aside the poultice; but I did not begin to lessen the dose of Opium till the 23d of May; after which time I went on gradually diminishing it, till the patient omitted it altogether; and on the 12th of June I discharged him from the hospital perfectly recovered; and eighteen months afterwards I had an opportunity of inquiring after him, and had the pleasure to find he had continued well.

*St. Alban's-Street,*

*December* 15, 1784.

